# Pulseless Electrical Activity Cardiac Arrest

**DOI:** 10.21980/J8Z055

**Published:** 2020-01-15

**Authors:** Erik Sembroski, Christopher M McDowell, Matthew M Mannion

**Affiliations:** * University of Missouri—Kansas City School of Medicine, Department of Internal Medicine, Kansas City, MO; ^ Southern Illinois University School of Medicine, Department of Emergency Medicine, Springfield, IL

## Abstract

**Audience:**

This simulation-based scenario is appropriate for senior level emergency medicine residents.

**Introduction:**

Pulseless electrical activity (PEA) accounts for up to 25% of sudden cardiac arrest;^1^ therefore the ability to recognize and care for this condition is an essential skill of emergency medicine physicians. Management of PEA arrest in the emergency department centers on Advanced Cardiac Life Support (ACLS) algorithms and the identification and treatment of potentially reversible causes. Massive pulmonary embolism (PE) is one of several causes of PEA cardiac arrest.^2^ However, diagnosis by CT-angiographic or nuclear imaging may not be obtainable in the hemodynamically unstable patient, requiring physicians to have a high index of suspicion. Systemic thrombolytic therapy is indicated in cardiac arrest due to known or presumed massive pulmonary embolism.^3^,^4^,^5^

**Educational Objectives:**

After competing this simulation-based session, the learner will be able to:

1. Identify PEA arrest
2. Review the ACLS commonly recognized PEA arrest etiologies via the H &T mnemonic
3. Review and discuss the risks and benefits of tissue plasminogen activator (tPA) for massive PE

**Educational Methods:**

This is a high-fidelity simulation that allows learners to evaluate and treat a PEA arrest secondary to massive PE in a safe environment. The learners will demonstrate their ability to recognize a PEA arrest, sort through possible etiologies, and demonstrate treatment of a massive PE with tPA. Debriefing will focus on diagnosis and management of the PEA arrest.

**Research Methods:**

This case was piloted with 12 PGY-2 and PGY-3 residents. Group and individual debriefing occurred post-case.

**Results:**

Post-simulation feedback from the faculty suggested two potential issues. First was fidelity, which we increased by using our ultrasound simulator. Second, the elevated presenting glucose with lactic acidosis could be a poor cue, leading some towards diabetic ketoacidosis (DKA).

**Discussion:**

Learners felt more confident about running a PEA arrest. The simulation improved resident awareness of the value of point of care ultrasound (POCUS) in cardiac arrest. It also clarified the dosing of tPA in massive PE. Faculty felt simulating the actual US without breaking simulation would be more challenging without our US simulator. Although there was concern about results pointing towards possible DKA, this did not occur in any of the pilot simulations. The presenting glucose was reduced to make this less likely in future simulations.

**Topics:**

Pulseless electrical activity (PEA), syncope, cardiac arrest, Hs and Ts from ACLS PEA instruction, tPA for massive PE, critical care medicine, simulation.

## USER GUIDE

[Table t1-jetem-5-1-s1]

**Table t1-jetem-5-1-s1:** 

List of Resources:	
Abstract	1
User Guide	3
Instructor Materials	5
Operator Materials	15
Debriefing and Evaluation Pearls	18
Simulation Assessment	21

**Learner Audience:**

Senior residents

**Time Required for Implementation:**

Instructor Preparation: 30–60 minutes

Time for case: 15–30 minutes

Time for debriefing: 15–30 minutes

**Recommended Number of Learners per Instructor:**

1

**Topics:**

Pulseless electrical activity (PEA), syncope, cardiac arrest, Hs and Ts from ACLS PEA instruction, tPA for massive PE, critical care medicine, simulation.

**Objectives:**

By the end of this simulation session, the learner will be able to:

1. Identify PEA arrest
2. Review the ACLS commonly recognized PEA arrest etiologies via the H &T mnemonic
3. Review and discuss the risks and benefits of (tPA) for massive PE

### Linked objectives and methods

By completing this simulation, learners will take a brief history and physical before the patient arrests. After intubation, the patient will arrest and cardiopulmonary resuscitation (CPR) will be initiated. The learner will identify PEA as the rhythm (objective 1) and continue the resuscitation while divulging the possible etiologies via the H&Ts mnemonic (objective 2). Once PE is recognized as a likely cause, the learner with discuss the pros & cons of tPA administration or be prompted to do so. Although learners are familiar with PE, most learners have not all seen cardiac arrest secondary to massive PE. Simulation develops comfort with diagnostic uncertainty and the administration of tPA without confirmatory imaging findings. This case forces them to process the tPA administration risk and benefits in a safe environment (objective 3). Debriefing will ensure that any potential misconceptions or knowledge gaps are addressed.

### Recommended pre-reading for instructor

- DePalo VA, Kharnaf AE. Venous Thromboembolism (VTE) Treatment & Management. In: Medscape. Panchbhavi VK, ed.
  https://emedicine.medscape.com/article/1267714-treatment#d10. Published June 12, 2019. Accessed July 2, 2019.
- Kline JA. Pulmonary embolism and deep vein thrombosis. In: Walls RM, Hockberger RS, Gausche-Hill M, et al. *Rosen’s Emergency Medicine: Concepts and Clinical Practice.* 9^th^ ed. Philadelphia, PA: Elsevier; 2018:1051–1066.
- Nickson C. Thrombolysis for submassive pulmonary embolus. Life in the Fast Lane.
  https://litfl.com/thrombolysis-for-submassivepulmonary-embolus/ Updated June 9, 2019. Accessed July 2, 2019.

### Results and tips for successful implementation

This case is best implemented with a team comprised of the learner/upper level resident, pharmacist, nurse, paramedic and family member. We recommend inviting your ED nurses, pharmacists or medics to participate. Faculty or other residents can act as family or embedded participants if staff is not available. The case was initially completed by 12 PGY-2 and PGY-3 residents. Verbal debriefing evaluating the simulation efficacy showed that learners became more comfortable initiating tPA without confirmatory imaging after completing this case. After the initial pilot, laboratory data (eg, venous blood gas, glucose) was amended to prevent exploration of diabetic ketoacidosis as potential etiology. Stimuli were modified to improve the image quality after case feedback.

## Supplementary Information











## Figures and Tables

**Table t2-jetem-5-1-s1:** 

#1	Electrocardiogram (ECG)
#2	Chest Radiograph (CXR)
#3	Ultrasound/Echocardiogram
#4	Computed Tomography (CT)
#5	Basic Metabolic Profile
#6	Lactic Acid
#7	Venous Blood Gas

**Table t3-jetem-5-1-s1:** 

**Vitals:**	Heart Rate (HR) 137	Blood Pressure (BP) 110/68	Respiratory Rate (RR) 23
	Temperature (T) 36.9°C	Oxygen Saturation (O_2_Sat) 76% on room air (RA)	

**Figure f1-jetem-5-1-s1:**
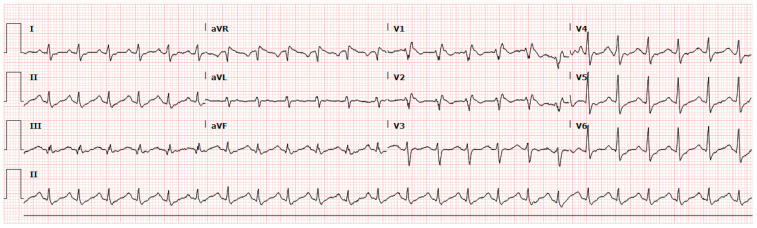


**Figure f2-jetem-5-1-s1:**
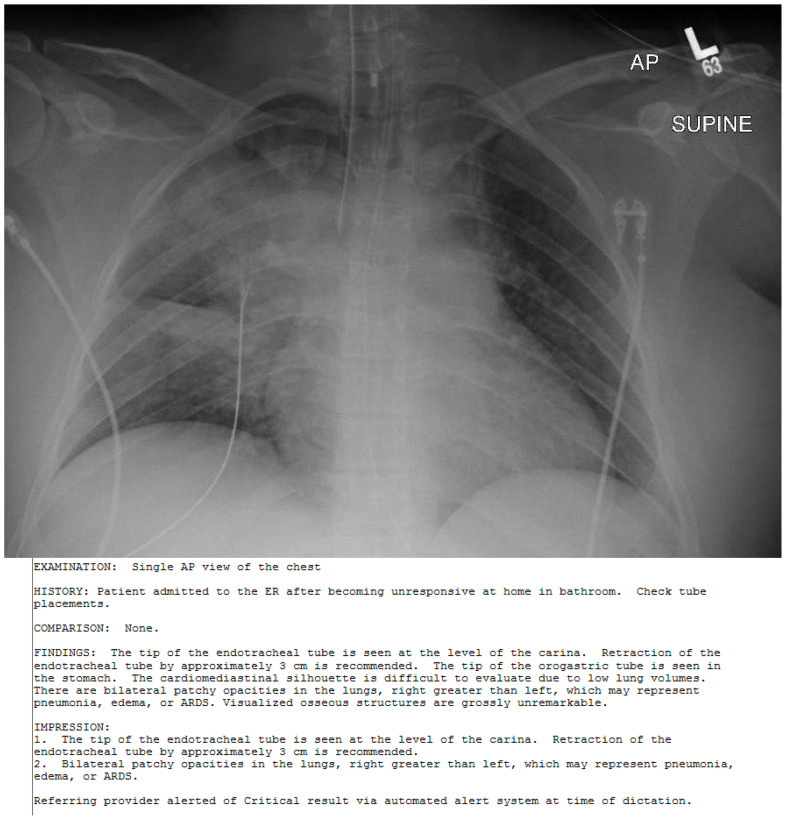


**Figure f3-jetem-5-1-s1:**
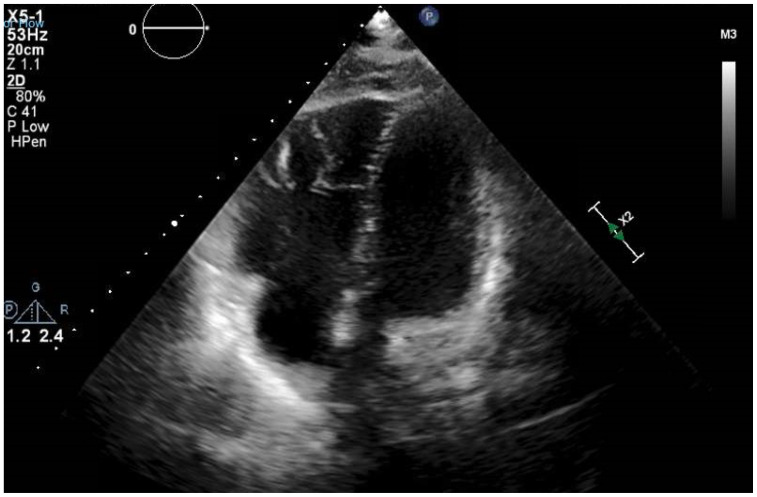


**Figure f4-jetem-5-1-s1:**
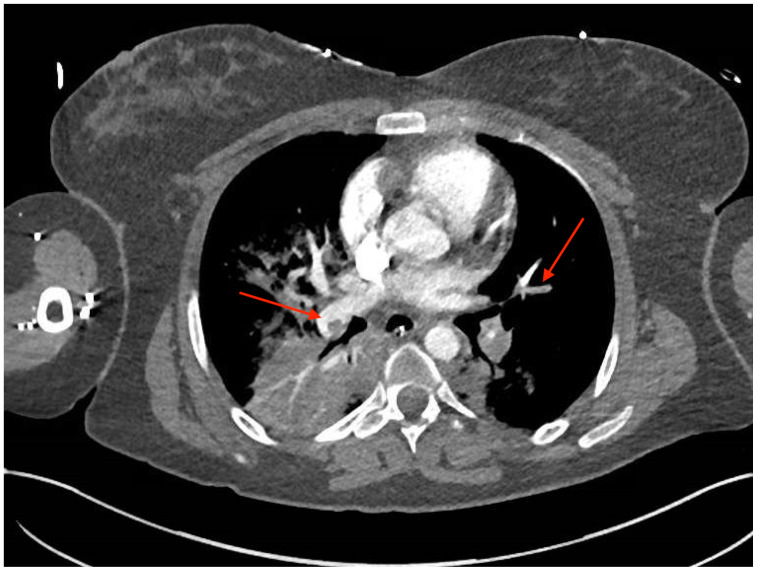


**Table t4-jetem-5-1-s1:** 

*Basic metabolic panel (BMP)*	
Sodium	140 mEq/L
Chloride	101 mEq/L
Potassium	3.6 mEq/L
Bicarbonate (HCO_3_)	18 mEq/L
Blood Urea Nitrogen (BUN)	13 mg/dL
Creatine (Cr)	1.1 mg/dL
Glucose	160 mg/dL
Ionized Calcium	4.4 mg/dL

**Table t5-jetem-5-1-s1:** 

*Lactic Acid*	4.1 mEq/L

**Table t6-jetem-5-1-s1:** 

*Venous Blood Gas (VBG)*	
pH	7.1
PaCO2	57 mmHg
PaO2	48 mmHg
Bicarbonate (HCO_3_)	16.9 mEq/L
O2 saturation, arterial	79%

**Table t7-jetem-5-1-s1:** SIMULATION EVENTS TABLE:

Minute (state)	Participant action/trigger	Patient status (simulator response) & operator prompts	Monitor display(vital signs)

0:00 (Baseline)	Participants enter the room.	Patient is lying on stretcher, agitated, tachypneic. Paramedics are at bedside to give report. Should be given in a casual/dismissive tone.	Not on monitor

01:00	Assess ABCs	A: Patient is alert, oriented to self, protecting her airway, speaking and moaning in pain.	T 36.9°C HR 137 BP 110/68 RR 23 O2 76% room air
	Place patient on monitor	B: Tachypneic, breathing in high 20s. In severe respiratory distress but with clear lung sounds.	
	Establish IV access	C: She is tachycardic, pale, mottled, but with adequate initial blood pressure.	
	Place patient on supplemental oxygen	When placed on supplemental O2 nonrebreather (NRB) patient is adamant it be removed, states “I can’t breathe.”	
	Attempt further history from EMS and patient, perform exam	She is able to say her name, but otherwise does not answer questions. Only states “it hurts” and “I can’t breathe.” Does not localize pain when asked.	

03:00 (Initial Resuscitation)	Begin IV fluids	Tachycardia and blood pressure will stabilize with IVF; however, she will not improve.	T 36.6°C HR 150 BP 96/50 RR 32 O2 88% NRB
	Oxygen supplementation	Patient continues to require increasing amount of supplemental oxygen.	
	Obtain ECG	ECG provided.	
	Obtain labs	Participants will be provided with labs if requested and should be prompted for them when IV is started if they do not request labs. Despite adequate resuscitation the patient’s condition continues to decline. Oxygenation worsens despite NRB and patient will not tolerate BiPAP. Tachycardia and hypoxia should continue to progress until decision is made to intubate.	
	Decision to intubate	Proceed to intubation state.	

05:00(Delay in treatment)	Despite IV fluids	Tachycardia increases and blood pressure decreases.	T 36.6°C HR 162 BP 84/41 RR 35 O2 75% nasal cannula
	Despite Oxygen supplementation	Hypoxia worsens.	
	No Decision to Intubate	Patient arrests—proceed to arrest state.	

05:00 Intubation	Prepare for intubation: - Suction - Bag-valve mask - Induction agent - Paralytic	Patient paralyzed and sedated.	T 36.6°C HR 162 BP 102/51 RR 12 O2 92% ventilator
	Patient should be reevaluated following intubation	Following intubation, patient’s HR decreases steadily into 70s. BP should be rechecked and is no longer obtainable. Proceed to arrest state.	T 36.6°C HR 74 BP– RR 12 O2 80% ventilator

10:00PEA Arrest	Begin CPR and progress through ACLS for treatment of PEA Arrest	Patient remains in PEA arrest with unchanged condition.	T 36.6°C HR PEA bagged BP– RR 12 O2 60% ventilator
	Discussion of Reversible causes (Hs and Ts)	Participants may need to be prompted for this discussion while progressing through ACLS. Disparity between patient’s presentation of tachypnea and respiratory acidosis/hypoxia should be discussed. Patient’s family member may be interviewed and will confirm that she is on birth control.	
	Administer tPA	Proceed to ROSC.	

15:00 No tPA Administered	Continue CPR, ACLs	If tPA is not given for presumed PE, patient will progress to asystole and family member will ask for team to “stop everything.”	
	Administer tPA	Proceed to ROSC.	

15:00 ROSC	Request for pressor infusion	Blood pressure stabilizes, HR increases.	T 36.6°C HR 96 BP 112/60 RR 12 O2 90% ventilator
	Obtain bedside US and CXR if not done so already	Provide CXR and US images as requested.	
	Call for CTA	Provide CT images and reports.	
	Initiate Heparin Infusion		

(Case Completion)	Call ICU for admission	ICU accepts the patient.	Case Ends

**Table t8-jetem-5-1-s1:** 

**Critical Actions:** Establish IV access, place patient on monitor, apply oxygen Recognize critically ill patient Establish definitive airway Recognize PEA arrest and performed ACLS Discuss reversible causes of PEA Establish diagnosis of PE Administer tPA Admit to ICU	0:00

**Table t9-jetem-5-1-s1:** 

	Milestone	Did not achieve level 1	Level 1	Level 2	Level 3
1	**Emergency Stabilization (PC1)**	□ Did not achieve Level 1	□ Recognizes abnormal vital signs	□ Recognizes an unstable patient, requiring intervention Performs primary assessment Discerns data to formulate a diagnostic impression/plan	□ Manages and prioritizes critical actions in a critically ill patient Reassesses after implementing a stabilizing intervention
2	**Performance of focused history and physical (PC2)**	□ Did not achieve Level 1	□ Performs a reliable, comprehensive history and physical exam	□ Performs and communicates a focused history and physical exam based on chief complaint and urgent issues	□ Prioritizes essential components of history and physical exam given dynamic circumstances
3	**Diagnostic studies (PC3)**	□ Did not achieve Level 1	□ Determines the necessity of diagnostic studies	□ Orders appropriate diagnostic studies. Performs appropriate bedside diagnostic studies/procedures	□ Prioritizes essential testing Interprets results of diagnostic studies Reviews risks, benefits, contraindications, and alternatives to a diagnostic study or procedure
4	**Diagnosis (PC4)**	□ Did not achieve Level 1	□ Considers a list of potential diagnoses	□ Considers an appropriate list of potential diagnosis May or may not make correct diagnosis	□ Makes the appropriate diagnosis Considers other potential diagnoses, avoiding premature closure
5	**Pharmacotherapy (PC5)**	□ Did not achieve Level 1	□ Asks patient for drug allergies	□ Selects an medication for therapeutic intervention, consider potential adverse effects	□ Selects the most appropriate medication and understands mechanism of action, effect, and potential side effects Considers and recognizes drug-drug interactions
6	**Observation and reassessment (PC6)**	□ Did not achieve Level 1	□ Reevaluates patient at least one time during case	□ Reevaluates patient after most therapeutic interventions	□ Consistently evaluates the effectiveness of therapies at appropriate intervals
7	**Disposition (PC7)**	□ Did not achieve Level 1	□ Appropriately selects whether to admit or discharge the patient	□ Appropriately selects whether to admit or discharge Involves the expertise of some of the appropriate specialists	□ Educates the patient appropriately about their disposition Assigns patient to an appropriate level of care (ICU/Tele/Floor) Involves expertise of all appropriate specialists
9	**General Approach to Procedures (PC9)**	□ Did not achieve Level 1	□ Identifies pertinent anatomy and physiology for a procedure Uses appropriate Universal Precautions	□ Obtains informed consent Knows indications, contraindications, anatomic landmarks, equipment, anesthetic and procedural technique, and potential complications for common ED procedures	□ Determines a back-up strategy if initial attempts are unsuccessful Correctly interprets results of diagnostic procedure
20	**Professional Values (PROF1)**	□ Did not achieve Level 1	□ Demonstrates caring, honest behavior	□ Exhibits compassion, respect, sensitivity and responsiveness	□ Develops alternative care plans when patients’ personal beliefs and decisions preclude standard care
22	**Patient centered communication (ICS1)**	□ Did not achieve level 1	□ Establishes rapport and demonstrates empathy to patient (and family) Listens effectively	□ Elicits patient’s reason for seeking health care	□ Manages patient expectations in a manner that minimizes potential for stress, conflict, and misunderstanding. Effectively communicates with vulnerable populations, (at risk patients and families)
23	**Team management (ICS2)**	□ Did not achieve level 1	□ Recognizes other members of the patient care team during case (nurse, techs)	□ Communicates pertinent information to other healthcare colleagues	□ Communicates a clear, succinct, and appropriate handoff with specialists and other colleagues Communicates effectively with ancillary staff

## INSTRUCTOR MATERIALS

**Case Title:** Pulseless Electrical Activity Cardiac Arrest

**Case Description & Diagnosis (short synopsis):** Patient is a 40-year-old female brought in by EMS for a suspected syncopal episode after being found unconscious. She was awake and alert at time of EMS arrival but grew increasingly agitated. Paramedics have grown frustrated with her agitation, and her agitation continues upon arrival. She will complain of pain but will not localize and grows progressively distressed. The patient is unable to provide further detailed history, though her family confirms she began birth control in the last few months. She is negative for any other PE risk factors. While in the emergency department she grows increasingly tachypneic, tachycardic, and hypoxic, eventually requiring intubation. Following intubation her blood pressure and pulse are lost and she has a PEA arrest. Participants will need to consider possible etiologies for PEA arrest and implement a treatment strategy. If Hs & Ts are not fully discussed, learners will be verbally prompted by an embedded participant. Participants should establish the diagnosis of massive PE. Ultrasound (US) confirms dilated right ventricle, and participants should discuss the risk and benefits of tPA administration. Embedded participant will prompt the tPA discussion if necessary as well. Participants should then administer tPA, after which they will obtain return of spontaneous circulation (ROSC). Participants may then order computed tomography (CT), which reveals massive PE with bilateral segmental emboli. If participants fail to follow ACLS guidelines, establish the diagnosis of PE, or fail to administer tPA, the patient will proceed to asystole and death.

**Equipment or Props Needed:**

High fidelity simulator

Infusion pump

IV pole

Cardiac monitor/defibrillator

Crash cart & medications

Intubation equipment

tPA

**Confederates needed:**

Nurse

Paramedic (control room)

Pharmacist (control room)

Family member (control room)

**Stimulus Inventory:** [Table t2-jetem-5-1-s1]

**Background and brief information:** The scenario occurs in the emergency department at a tertiary care center. The patient is a 40-year-old female brought in by EMS for a suspected syncopal episode. The patient was found unconscious on bathroom floor at work by coworker. She was awake and alert at time of EMS arrival but grew increasingly agitated. Paramedics have grown frustrated with her agitation and refusal to cooperate with their assessment, and this continues on their arrival. She will complain of pain but will not localize and grows progressively distressed in the emergency department. The patient is unable to provide further detailed history, though her family will confirm that she began birth control in the last few months. She is negative for any other PE risk factors. While in the emergency department she grows increasingly tachypneic, tachycardic, and hypoxic, eventually requiring intubation. Following intubation her blood pressure and pulse are lost and she has a PEA arrest. Participants will need to consider possible etiologies for PEA arrest and implement a treatment strategy. If Hs & Ts are not fully discussed, learners will be verbally prompted by an embedded participant.

**Initial presentation:** Patient is brought in by EMS, who appear frustrated with the patient’s lack of cooperation. She is alert, but in severe distress. She is oriented to self but otherwise minimally able to cooperate with history and exam. She is tachypneic and complains of pain and discomfort but cannot localize cause of her distress. She is very agitated, attempting to remove her gown and oxygen mask.

**How the scenario unfolds:** This patient has a massive PE. Paramedics will be present at the beginning of the case and will give her presenting history in a calm, dismissive tone. The participants should recognize that the patient is sicker than this report suggests and establish IV access, place the patient on the monitor, and apply oxygen. Laboratory and bedside imaging (CXR/US) may be requested at this time, but participants will be told the patient is too unstable for CT. Other than the agitation, tachycardia, and respiratory distress, the exam is largely unremarkable. The patient quickly grows increasingly distressed, tachypneic and tachycardic and eventually hypoxic. If the participants do not elect to intubate early, the patient will become progressively hypoxic until this decision is reached (if they attempt to place BiPAP, they will be told that the patient tears it off). Following intubation, the patient has a rapid decline in blood pressure, eventually resulting in loss of pulses and a PEA arrest. The participants should then verbalize Hs and Ts or another approach to determining reversible causes of PEA arrest. The patient will remain in PEA arrest as long as the participants follow ACLS. The participants should identify a massive PE as the most likely cause of the arrest and administer tPA (100 mg IV over two hours). The patient will then have ROSC. Bedside US should be available and if asked for will show right ventricular dilation. Following ROSC and stabilization, if the participants request a CT this will show PE with bilateral segmental emboli. The learners should then discuss with the ICU for admission.

**Critical actions:**

1. Establish IV access, place the patient on the monitor, and apply oxygen
2. Recognize critically ill patient
3. Intubate
4. Recognize PEA arrest and perform ACLS
5. Discuss reversible causes of PEA
6. Establish diagnosis of PE
7. Administer tPA
8. Admit to ICU

**Case Title:** Pulseless Electrical Activity Cardiac Arrest

**Chief Complaint:** Arrives via EMS. Found in bathroom pale after possible syncope vs. seizure.[Table t3-jetem-5-1-s1]

**General Appearance:** 40-year-old female who appears agitated, mottled and dyspneic.

**Primary Survey:**

- **Airway:** moaning and making incomprehensible noises but not able to state her name when asked
- **Breathing:** rather tachypneic, increased work of breathing
- **Circulation:** pale, cool extremities; no palpable distal pulses

**History:**

- **History of present illness:** 40-year-old female presents in acute respiratory distress. Per EMS she was found on the bathroom floor by a family member after a loss of consciousness. Initially, they were called for a possible seizure, but on arrival, the patient was agitated and combative. She reports pain and severe trouble breathing but is unable to localize her pain. Her family member arrives and provides history that the patient is on birth control.
- **Past medical history:** Attention deficit disorder
- **Past surgical history:** none
- **Patient’s medications:** oral contraceptive pills, amphetamine 20mg
- **Allergies:** No known allergies (NKDA)
- **Social history:** negative for tobacco or recreational drug usage. Occasional alcohol consumption
- **Family history:** unremarkable

**Secondary Survey/Physical Examination:**

- **General appearance:** patient appears tachypneic and in significant respiratory distress
- **HEENT:**
  ○ **Head:** normocephalic, atraumatic
  ○ **Eyes:** Pupils equal and reactive bilaterally
  ○ **Ears:** Within normal limits (WNL)
  ○ **Nose:** WNL
  ○ **Throat:** WNL
- **Neck:** WNL
- **Heart:** tachycardic, no murmurs/rubs/gallops
- **Lungs:** diminished but present lung sounds bilaterally; no rales. Severe respiratory distress noted.
- **Abdominal/GI:** soft, mild diffuse tenderness to palpation; no guarding/rebound
- **Genitourinary:** N/A
- **Rectal:** N/A
- **Extremities:** WNL
- **Back:** WNL
- **Neuro:** alert to self only, otherwise uncooperative
- **Skin:** pale/mottled
- **Lymph:** WNL
- **Psych:** agitated, barely conscious

**Results:**

*Electrocardiogram (ECG)*

*(author’s own image)* [Fig f1-jetem-5-1-s1]

*Chest Radiograph (CXR)*

*(author’s own image)*

*If chest x-ray is ordered early in the case, learners will be informed it is on the way. CXR can be taken following intubation, arrest, and tPA and is shown below.* [Fig f2-jetem-5-1-s1]

*Ultrasound/Echocardiogram*

*(author’s own image)* [Fig f3-jetem-5-1-s1]

*Computed Tomography (CTA)*

*(author’s own image)*

*Following arrest, tPA, resuscitation, and ROSC, a CTA may be requested, with image below:* [Fig f4-jetem-5-1-s1]

Impression:

1. Extensive pulmonary embolism as described with right ventricular strain
2. Large areas of consolidation in the right lung and to a lesser degree in the left lower lobe indicating pneumonia
3. Periportal edema in the liver and mild ascites
4. Fluid surrounding the pancreas suspicious for acute pancreatitis
5. A 3 cm gastric mass in the distal stomach suspicious for a neoplasm[Table t4-jetem-5-1-s1][Table t5-jetem-5-1-s1][Table t6-jetem-5-1-s1]

*If further labs are requested, the learners should be informed that lab is running them.*

## OPERATOR MATERIALS

[Table t7-jetem-5-1-s1]

### Diagnosis

Massive PE; PEA arrest with ROSC

### Disposition

Admission to the medical ICU.

## DEBRIEFING AND EVALUATION PEARLS

### PEA Arrest

**Pearls:**

**Reversible Causes:**

**Hs and Ts**

- Hypovolemia, hypoxia, hydrogen ions (acidosis), hypokalemia, hyperkalemia, Hypoglycemia, Hypothermia
- Toxins, tamponade (cardiac), tension pneumothorax, thromboembolism (pulmonary or cardiac)

**Alternate Approach**

Fix with airway

- Hypoxia
- Acidosis (if respiratory)

Fix with a needle

- Tension pneumothorax
- Cardiac tamponade

Fix with an IV

- Hypovolemia – fluid, blood
- Hyperkalemia – calcium, bicarbonate, insulin
- Hypokalemia – potassium/magnesium
- Acidosis (if metabolic) – bicarb
- Hypoglycemia - dextrose
- Massive PE – tPA
- Toxins – antidote

Fix with Dialysis

- Hyperkalemia
- Acidosis
- Toxins

Fix with warming

- Hypothermia

Fix with tPA or heart catheterization

- Acute myocardial infarction

**ECG findings of PE**

- S1Q3T3 associated with PE (S1Q3 seen on case ECG)
- Most commonly sinus tachycardia
- Signs of Right heart strain including Right bundle branch block

**Ultrasound Findings**

- Right ventricular dilation
- Hyperdynamic cardiac function
- McConnell’s sign (relative hyperkinesis of apex of right ventricle)

**Other debriefing points:**

- Discuss the index of suspicion created on entering the room. Report by EMS and their causal air in contrast to patient’s appearance. How quickly was safety net established and did initial vitals change participant’s perception of the case?
- Discuss the treatment and evaluation of PEA arrest, including progression through analyzing reversible causes and what factors change index of suspicion for each one.
- Discuss the dosing of tPA in massive PE.
- Why did this patient arrest after intubation? (blunting catecholamine response)
- If bedside US was not obtained, discuss how else this patient’s likelihood of PE could have been assessed.
- What are this patient’s risk factors for PE vs other causes of PEA? What about her presentation suggested PE?

## SIMULATION ASSESSMENT

*PEA Arrest*

Learner: _________________________________________

***Assessment Timeline***

This timeline is to help observers assess their learners. It allows observer to make notes on when learners performed various tasks, which can help guide debriefing discussion.[Table t8-jetem-5-1-s1]

**Critical Actions:**

□ Established IV access, place patient on monitor, apply oxygen
□ Recognized critically ill patient and began resuscitation early
□ Established definitive airway
□ Performed ACLS and recognized PEA arrest
□ Discussed reversible causes of PEA
□ Establish diagnosis of PE
□ Administer tPA
□ Patient admitted to ICU

**Summative and formative comments:**

**Milestones assessment:** [Table t9-jetem-5-1-s1]
