# Supplementary figures and images for: Pulseless Electrical Activity Cardiac Arrest
Source: J Educ Teach Emerg Med. 2020 Jan 15;5(1):S1–S25. doi: 10.21980/J8Z055 (PMC10332535; doi:10.21980/J8Z055)

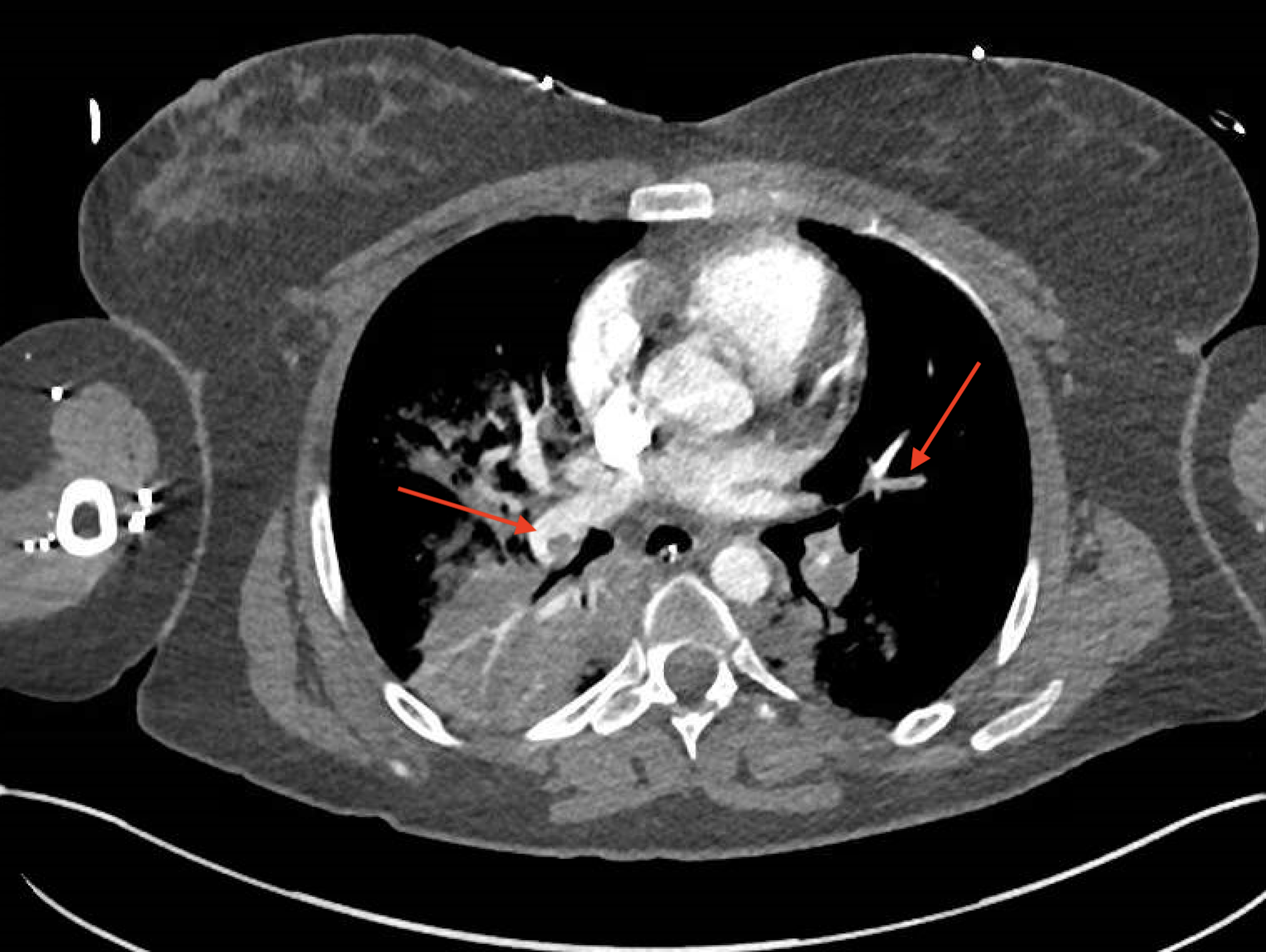

Supplement: Supplementary file 1 [file jetem-5-1-s1-supp1.jpg]

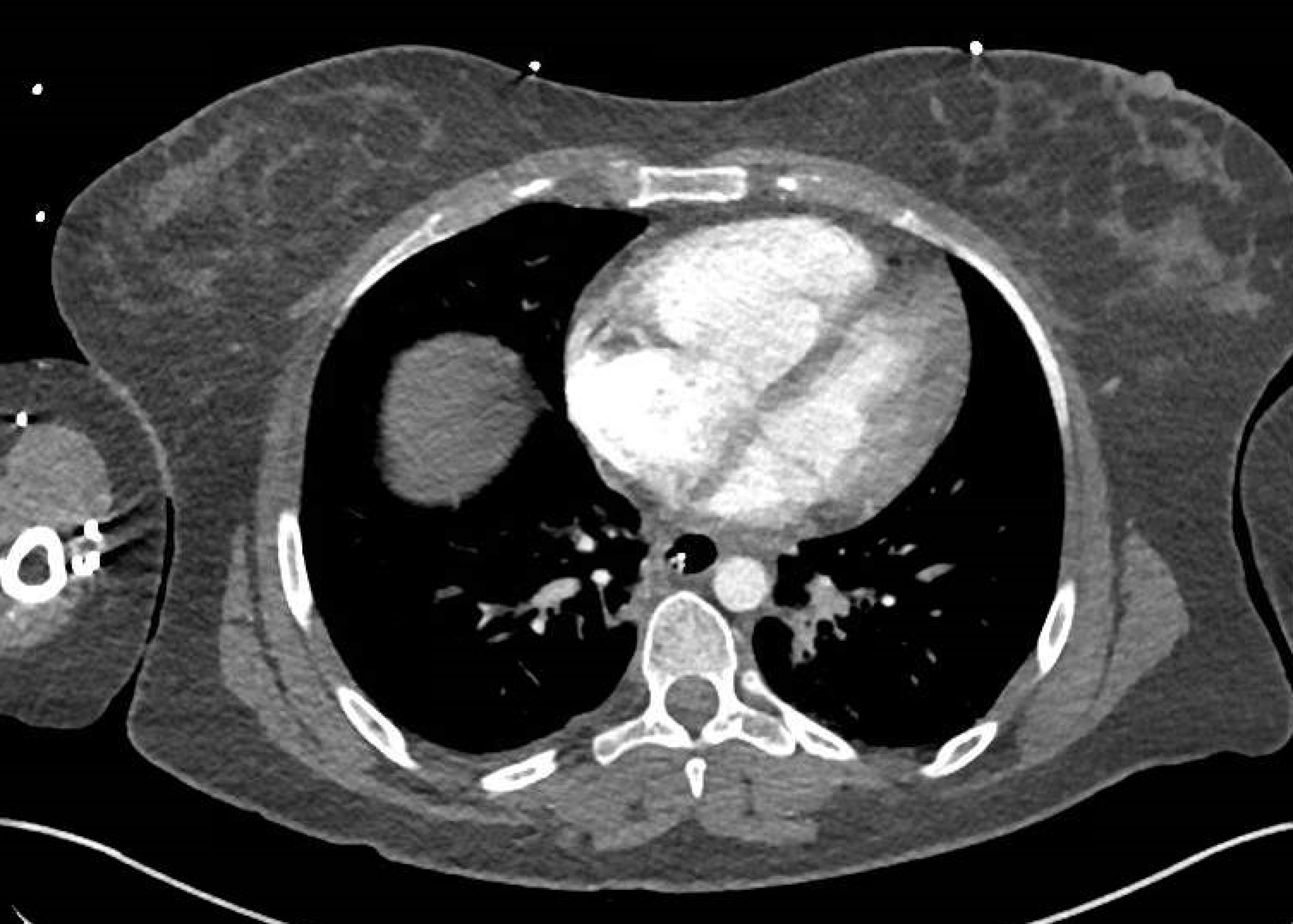

Supplement: Supplementary file 2 [file jetem-5-1-s1-supp2.jpg]

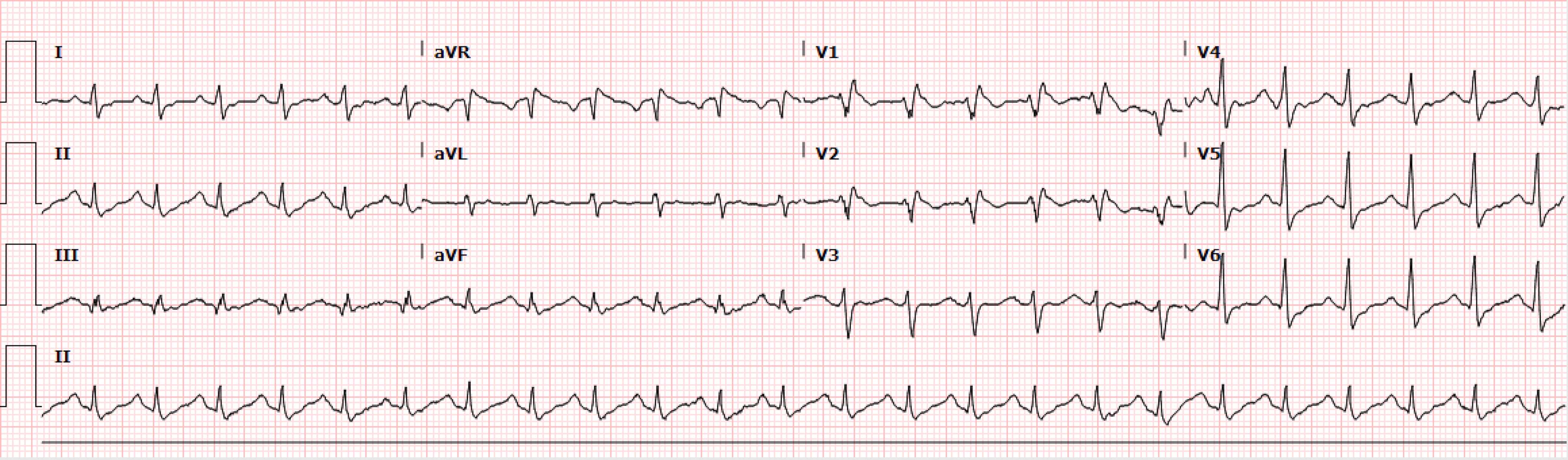

Supplement: Supplementary file 4 [file jetem-5-1-s1-supp4.jpg]

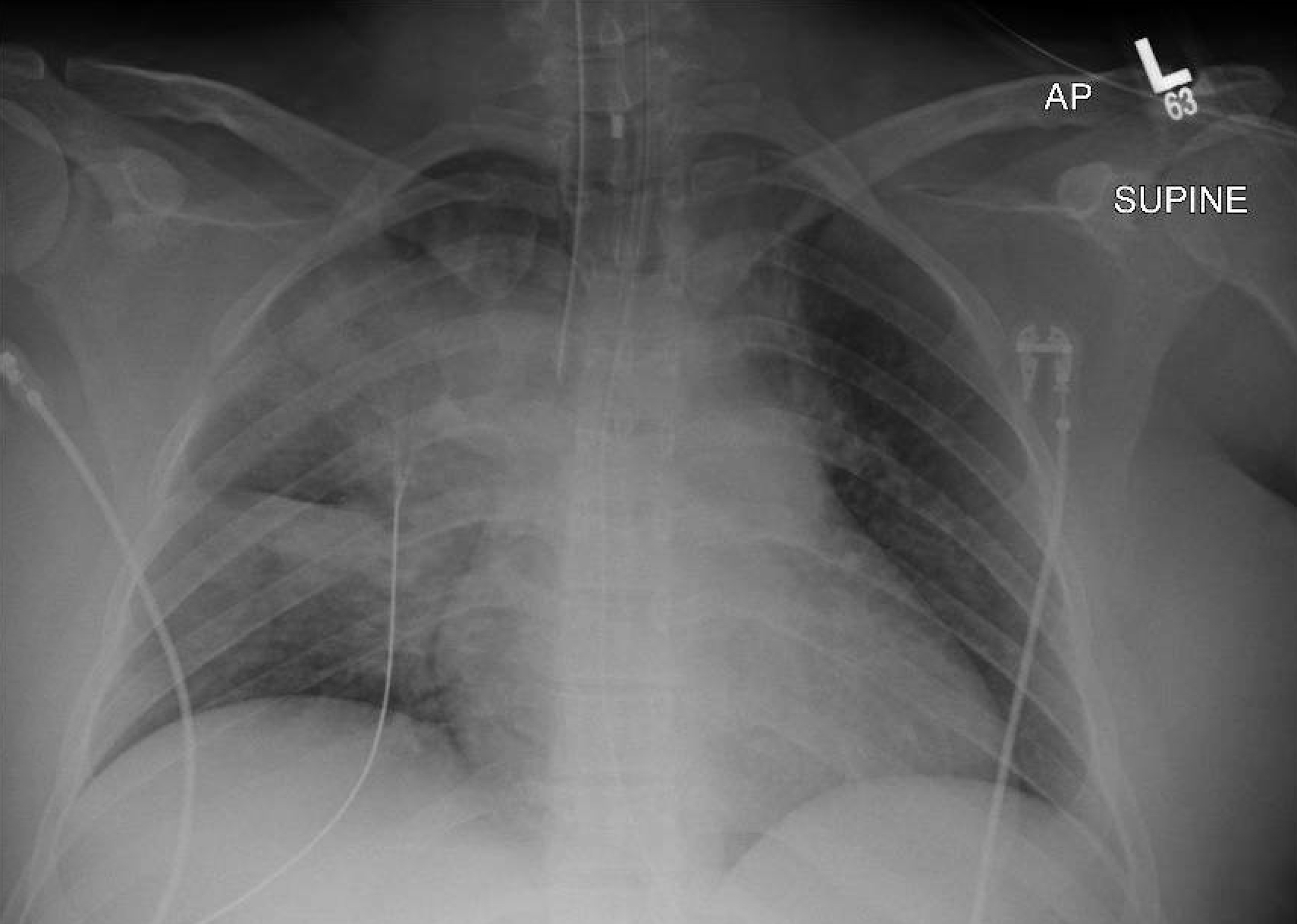

Supplement: Supplementary file 5 [file jetem-5-1-s1-supp5.jpg]
